# Cumulative Effect of Depression on Dementia Risk

**DOI:** 10.1155/2013/457175

**Published:** 2013-09-12

**Authors:** J. Olazarán, R. Trincado, F. Bermejo-Pareja

**Affiliations:** ^1^Alzheimer Disease Research Unit, Alzheimer Center Reina Sofia Foundation—CIEN Foundation, Carlos III Institute of Health, Madrid, Spain; ^2^Neurology Service, Gregorio Marañón General University Hospital, Madrid, Spain; ^3^Neurology Service, 12 de Octubre University Hospital, Madrid, Spain; ^4^Program 1—Alzheimer's Disease and Other Degenerative Dementias, CIBERNED, Spain

## Abstract

*Objective*. To analyze a potential cumulative effect of life-time depression on dementia and Alzheimer's disease (AD), with control of vascular factors (VFs). *Methods*. This study was a subanalysis of the Neurological Disorders in Central Spain (NEDICES) study. Past and present depression, VFs, dementia status, and dementia due to AD were documented at study inception. Dementia status was also documented after three years. Four groups were created according to baseline data: never depression (nD), past depression (pD), present depression (prD), and present and past depression (prpD). Logistic regression was used. *Results*. Data of 1,807 subjects were investigated at baseline (mean age 74.3, 59.3% women), and 1,376 (81.6%) subjects were evaluated after three years. The prevalence of dementia at baseline was 6.7%, and dementia incidence was 6.3%. An effect of depression was observed on dementia prevalence (OR [CI 95%] 1.84 [1.01–3.35] for prD and 2.73 [1.08–6.87] for prpD), and on dementia due to AD (OR 1.98 [0.98–3.99] for prD and OR 3.98 [1.48–10.71] for prpD) (fully adjusted models, nD as reference). Depression did not influence dementia incidence. *Conclusions*. Present depression and, particularly, present and past depression are associated with dementia at old age. Multiple mechanisms, including toxic effect of depression on hippocampal neurons, plausibly explain these associations.

## 1. Introduction

Depression has been associated with an increased risk of dementia in old age, but the mechanisms underlying this association are not well understood. Several hypothetical, not mutually exclusive, mechanisms can be proposed: (1) depression is a prodrome of dementia, (2) depression reduces the threshold for dementia manifestation, and (3) depression leads to damage to neural systems, particularly the hippocampus, thus contributing to the development of dementia [1]. In addition, depression can be regarded as a potential reaction to early cognitive deficits. In fact, one epidemiological study reported depressive symptoms following the onset of dementia, rather than preceding it [2]. Under any of the above-mentioned mechanisms, depression at any time during life should be associated with an increased risk of dementia in unselected old population. 

An important role of vascular factors (VFs) in the association between depression and dementia is also possible. Indeed, previous studies demonstrated that cerebrovascular disease (CVD), hypertension, diabetes, and other VFs may cause both cognitive impairment [3, 4] and depression [5]. Depression could also influence platelet aggregation and vascular reactivity, leading to CVD and dementia [6]. However, either causal or mediator, this potential role of CVD and other VFs in the association between depression and dementia has barely been investigated [7]. 

We analyzed data from an epidemiological survey to ascertain whether the occurrence of depression across lifetime predicts dementia in the old age. To elucidate the involved mechanisms, the effects of past depression, present depression, and both were analyzed separately, with rigorous control of the VFs. Hypothetically, neural damage caused by direct toxicity of depression should lead to a cumulative effect of past and present depression on dementia risk, independently of VFs. 

## 2. Methods

### 2.1. The NEDICES Study

Data were derived from the Neurological Disorders in Central Spain (NEDICES) study, a longitudinal, population-based survey of major age-associated neurological conditions in people aged 65 or over who lived in three communities (one rural, i.e., the Arévalo District, Ávila and two urban, i.e., Getafe and Lista, Madrid). A detailed account of the study population, sampling methods, and assessments was published elsewhere [8, 9]. The NEDICES cohort survey completed two cross-sectional waves, with May 1, 1994 (basal) and May 1, 1997 as the prevalence dates. Each wave comprised two phases. In phase I (screening), a structured interview was conducted that contained questions regarding sociodemographic data, lifestyle, current medications, and several screening questions aimed at detecting medical, psychiatric, and neurological conditions. An adapted Minimental State (MMS) [10] and a functional scale [11] were also administered at that phase. Assessments were conducted by trained interviewers who gathered information from the subject alone or, when available, from an informant. 

In phase II, persons who screened positive for both the MMS and the functional scale were visited by one neurologist who addressed dementia diagnosis in accordance with DSM-IV criteria [12]. Should the dementia diagnosis was confirmed, the aetiology of dementia was established on the basis of interview, neurological examination, and medical records consultation. For the present investigation, dementia aetiology was divided in the three following categories: AD (i.e., probable or possible AD, without vascular component) [13], dementia with vascular component (DVC) (i.e., AD with evidence of CVD and vascular dementia) [14], and other dementia.

### 2.2. Assessment of VFs and Depression

The phase I structured interview of the NEDICES study included questions regarding medical diagnoses of hypertension, diabetes, heart disease, hypercholesterolemia, stroke (transient ischemic episodes included), and current medications. In case of use of medications related to the target diseases (e.g., antihypertensive drugs), the corresponding diseases were endorsed (e.g., hypertension). Current and past tobacco use were also documented and considered as VFs for the present investigation, whereas subjects with a positive history for (either present or past) alcohol abuse were eliminated from this study. 

In one of the study sites (i.e., Getafe), the phase I structured interview included questions regarding past depressive episodes and present depression. Past depressive episodes were documented using the following question: “have you ever suffered from depression (depressive disorder)?” There were three possible answers to this question: no, yes, and yes more than once. For those subjects who answered affirmatively, the dates of the episodes were requested, up to a maximum of three episodes. For those subjects from the target census population for whom the structured interview could not be conducted, a short questionnaire was designed and sent. In case of lack of answer, a shorter questionnaire was sent to the subject family physician. These questionnaires included one question regarding present depression, other question regarding past depressive episodes that required treatment, and, in the case of the subjects questionnaire, current medications. 

Subjects were included in the present study if information on both present and past depression was available, either from structured interview or short questionnaire. Present depression was endorsed according to answer to that question or, in case of structured interview, when there was a depressive episode in the last 10 years. This rather conservative long period was chosen because, for the present investigation, it was essential to identify true episodes of past depression to test a potential cumulative effect of depression episodes on dementia. Present depression was also endorsed if the subject was taking antidepressant medication, irrespective of his/her answer to the other questions. 

Four study groups were created following a hypothetical increasing risk of dementia: never depression (nD), past depression (pD), present depression (prD), and present and past depression (prpD). This hypothetical risk was derived from a proposed model of several additive pathways that would explain the association between depression and dementia [1, 2].

### 2.3. Statistical Analyses

Differences in proportions were assessed by means of *χ* ^2^ or Kruskall-Wallis tests, while differences in continuous variables across levels of categorical variables were tested using *t* test or analysis of variance (ANOVA). In the ANOVA analyses, between-group differences were explored using the Bonferroni test. Logistic regression models were conducted to examine whether depression was associated with dementia at baseline and at three-year follow-up assessments. The regression analyses were performed on two different scenarios: first, including all dementia cases; second, including only dementia due to AD (without vascular component). All the regression models were adjusted for the effects of age, sex, education, and VFs. The Hosmer and Lemeshow test was used to evaluate model fitness. Type I error for statistical significance was fixed at 0.05. Statistical analyses were performed using the SPSS 11.0 software (SPSS Inc., Chicago, Illinois, USA).

## 3. Results

The target census population totaled 5,278 individuals (89.2% of the original eligible census population). Thirteen subjects were eliminated due to alcohol abuse. Complete information on depression was not available in 3,458 of the remaining subjects, who mostly belonged to two (i.e., Arévalo and Lista) of the three study sites. This was so because, as mentioned in the methods section, an abridged protocol was used at those sites. Therefore, data of 1,807 subjects (81.9% from Getafe, 15.3% from Arévalo, and 2.8% from Lista) were analyzed in the present study. Compared to those subjects without complete information about depression, subjects with information had a lower educational achievement, a higher frequency of diabetes and hypercholesterolemia, lower frequency of tobacco use, and a more frequent diagnosis of dementia (Table 1).

There was a small predominance of women in the final sample (59.3%), which displayed low educational achievement (67.3% of the subjects had not completed primary school) (Table 1). A total of 121 subjects (6.7%) had dementia at the baseline assessment, with AD as the most frequent aetiology (71.4%). A vascular component in the aetiology of dementia was established in 16 subjects (13.2%). Most of the subjects (81.4%) did not endorse either present or past depression; past depression was documented in 4.7% of the subjects; present depression was documented in 10.2% of the subjects; both present and past depression appeared in 3.7% of the final sample. 

The demographic and clinical characteristics of the four study groups are shown in Table 2. Women were more represented in all the depression groups; in addition, the prpD group displayed less frequency of illiteracy and more frequency of subjects with primary school. There was also an uneven distribution of VFs across the depression groups: diabetes, hypercholesterolemia, and hypertension were more prevalent in the groups of depression, whereas smoking was more frequently reported in those subjects who never had depression. Past cerebrovascular episodes were particularly reported in the group of prpD (12.1%), which also presented the highest frequency of dementia diagnosis (13.6%). Remarkably, dementia due to AD was particularly frequent in the prpD group (88.9%) (Table 2).

Data on dementia status at three-year follow-up assessment could be obtained from 1,376 subjects (81.6% of the subjects who had not dementia at baseline). There were 86 cases of incident dementia (79.1% AD, 10.5% DVC, and 10.5% other dementia), that seemed to be evenly distributed across the four study groups: 76 subjects (5.5% of the subjects without dementia at baseline) in the nD group, 2 subject (2.5%) in the pD group, 6 subjects (3.6%) in the prD group, and 2 subjects (3.5%) in the prpD group (*P* > 0.05).

Information on tobacco use was missing in 447 subjects. Since preliminary analyses did not demonstrate a significant effect of tobacco on dementia prevalence or incidence, in order to achieve more statistical power, this variable was not included in the subsequent regression analyses. The final regression models demonstrated a significant effect of depression group in the prevalence of dementia at baseline (Table 3), with higher dementia prevalence in prD and prpD groups. Cerebrovascular episodes, but not other VFs, also predicted dementia. These results remained essentially unaltered when only dementia due to AD (without vascular component) was considered, although statistical significance was not achieved for the prD group, probably due to the small sample size. These results did not change when the total number of VFs, rather than every single factor, was introduced in the regression model (data not shown). Only old age and cerebrovascular episodes predicted dementia at the three-year follow-up assessment (regression analyses on dementia incidence are not shown). 

## 4. Discussion

We analyzed the effect of depression episodes across life time on the prevalence and incidence of dementia in a lowly educated cohort of people aged 65 or over. Present depression and, particularly, present and past depression were associated with concomitant dementia, but not with incidence of dementia after a follow-up period of three years. The results remained essentially unaltered once VFs were controlled and when only “pure” AD dementia (i.e., AD without vascular component) was analyzed. In the light of these results, a vascular mechanism to explain the association between depression and dementia seems very unlikely. The obtained results are consistent with previously reported data from a survey where highly educated subjects, 65 years of age or older, were analyzed. In that study, symptoms of depression at baseline were associated with an increased risk of mild cognitive impairment (MCI) after careful adjustment of vascular disease measures [7]. As genuine contributions from the present investigation, a sample of unusually low educational achievement was analyzed, depression episodes were recorded across life time, and the effect of depression on pure AD was additionally investigated. 

Past depression alone was not associated with dementia in the present investigation (Table 3), which was somewhat against the initial expectations. A recently published study found that late-life (i.e., ≥50 years), but not early-life (i.e., <50 years) depression, was associated with dementia [15]. That finding could be interpreted on the basis of a progressive increase in vulnerability, or loss of regeneration/plasticity, of hippocampal neurons during adulthood. Regretfully, methodological limitations did not permit us to delimit the duration of past depression episodes and hence analyze the potential differences derived from depression episodes suffered in early versus late adulthood. Nevertheless, risk of dementia derived from depression episodes suffered late in adulthood should be cautiously interpreted, since on that scenario, depression could be an early manifestation of, or a psychological reaction to, the neurodegenerative process that precedes dementia [1, 2]. 

As the most relevant finding from the present investigation, a significant increase in dementia prevalence was observed in the subjects for whom both present and past depression were documented (Table 3). The involved mechanisms for this association are probably multiple. Present depression may reduce the threshold for dementia manifestations, may represent a dementia prodrome, and may be a psychological reaction to the cognitive symptoms of dementia [1, 2]. These not mutually exclusive mechanisms clearly explain the association of present depression and prevalent dementia. In addition, the strong association observed between present and past depression and dementia prevalence supports a mechanism of direct and cumulative toxic effect of depression on brain neurons. 

The association of present and past depression and dementia was particularly high for dementia due to AD (OR 3.98, CI 95% 1.48–10.71, *P* < 0.005) (Table 3), which supports a specific mechanism of depression-induced damage to the hippocampal neurons [1]. A theory of glucocorticoid-mediated damage to the hippocampus was proposed to explain the memory loss that was observed in depression and other clinical conditions such as cushing syndrome and posttraumatic stress disorder [16]. Regardless of the involved chemical mediators, a “dose-dependent” effect should be observed under the assumption of neural toxicity caused by depression. In a recent study, depressive symptoms were prospective and periodically measured in 1,239 adults, and the influence of depressive symptoms on incident dementia was analyzed after a median follow-up period of 25 years. A monotonic increase in dementia risk of 14% was found for each episode of elevated depressive symptoms [17]. The apolipoprotein E (apoE) is involved in neural growth and repair, and the isoform apoE4 clearly increases the risk for developing AD [18]. In a cohort of 1,932 Japanese American men, individuals with depressive symptoms and the *APOEε*4 allele had a markedly increased risk of dementia [19]. Overall, these results suggest that depression, via toxic effect on neurons and failure of neural repair mechanisms, is an important contributor to AD dementia in predisposed subjects. Indeed, amyloid deposition and AD could be regarded as a common physiopathological pathway where different causes of neural toxicity may converge [20]. 

Yet other models could explain the observed associations of depression, dementia, and AD. It was documented that people who developed dementia at old age presented lower linguistic [21] and mental [22] capacities early in life, compared to people who did not develop dementia. It could be speculated that poor innate or acquired cognitive capacities may condition a poor coping style, leading to depressive episodes across lifetime. On the same vein, the association of depression and dementia could be explained on the basis of innate or acquired differences in the biological characteristics of the brain (i.e., brain reserve). However, the selective association between depression and dementia due to AD is hard to fit within these models. There is also a possibility that recurrent depression is a different disease than one single depressive episode in terms of neurobiology and genetics, which would explain the strong association between frequent depressive episodes and dementia and even the association between recurrent depression and AD. Future studies that include biological markers and analyze the effect of antidepressant pharmacological treatment may help to further clarify the physiopathological links of depression and dementia. 

The present study failed to demonstrate an association between depression and incidence of dementia, in spite of the fact that most previous studies demonstrated an increased risk of dementia in old subjects who endorsed depressive symptoms [17, 23–25]. In fact, the previous negative studies had small sample size [2, 26] or were not population based but performed in specialized clinical setting [27]. In addition, the follow-up periods were usually larger in the positive studies [17, 25]. Probably, our study failed to demonstrate association between depression and future dementia due to the reduced follow-up period and the small sample size. 

The present study had several other limitations. Depression data were only available in subjects who had less educational attainment and different VFs profile, compared to the original cohort (Table 1). In addition, life-time history of depression was retrospectively collected, and present depression was based on screening questions and medication, and this methodology differed across study sites. Nevertheless, our results confirmed the association of depression and AD, independently of VFs, even in such lowly educated population where some VFs were overrepresented.

## 5. Conclusions

Present depression and, particularly, present and past depression are associated with dementia at old age. These results hold for AD dementia and also after rigorous control of vascular risk factors and cerebrovascular disease. Multiple mechanisms, including toxic effect of depression on hippocampal neurons and innate or acquired predisposition for both dementia and depression, plausibly explain these results.

## Figures and Tables

**Table 1:** Characteristics of subjects with and without complete information on depression.

	Subjects with information (*n* = 1,807)	Subjects without information (*n* = 3,458)	*P* value
Age (mean, SD)	74.3 (7.0)	74.4 (7.0)	NS
Women	59.3	56.7	NS
Education			<0.001
Illiterate	22.4	8.9	
Can read and write	44.9	37.4	
Primary school	22.9	38.1	
≥secondary school	9.8	15.5	
Hypertension	52.2	50.5	NS
Diabetes	19.8	15.1	<0.001
Heart disease	9.7	10.3	NS
Hypercholesterolemia	32.7	27.0	<0.001
Tobacco use	35.7	40.1	<0.01
Stroke	5.3	4.6	NS
Dementia at baseline	6.7	5.3	<0.05
Dementia aetiology			
AD	71.4	72.4	NS
DVC	13.4	17.2	
Other aetiology	15.1	10.4	

Data represent % unless “mean, SD” is indicated.

AD: Alzheimer's disease (without vascular component); DVC: dementia with vascular component; NS: statistically not significant (*P* > 0.05).

**Table 2:** Characteristics of subjects in the four study groups.

	nD	pD	prD	prpD	*P* value
	(*n* = 1,471)	(*n* = 85)	(*n* = 185)	(*n* = 66)	
Age (mean, SD)	74.4 (7.1)	72.8 (6.2)	74.2 (6.3)	73.5 (6.9)	NS
Women	55.1	75.3	76.8	81.8	<0.001
Education					<0.001
Illiterate	22.3	22.4	27.6	10.6	
Can read and write	45.3	41.2	45.9	37.9	
Primary school	22.3	29.4	16.2	47.0	
≥Secondary school	10.1	7.1	10.3	4.5	
Hypertension	50.9	58.3	57.9	57.6	NS
Diabetes	18.4	22.4	27.2	25.8	<0.05
Heart disease	10.1	2.4	12.0	4.6	<0.05
Hypercholesterolemia	30.6	42.7	39.8	48.3	<0.005
Tobacco use^1^	38.8	22.2	22.0	18.8	<0.001
Stroke	4.9	3.5	7.0	12.1	<0.05
Dementia	6.0	7.1	9.7	13.6	<0.05
Dementia aetiology					<0.05
AD	74.7	20.0	61.1	88.9	
DVC	11.5	60.0	11.1	11.1	
Other aetiology	13.8	20.0	27.8	0.0	

Data represent % unless otherwise indicated.

^1^This variable was missing in 447 subjects.

nD: never depression; pD: past depression; prD: present depression; prpD: present and past depression; AD: Alzheimer's disease (without vascular component); DVC: dementia with vascular component; NS: statistically not significant (*P* > 0.05).

**Table 3:** Final regression model for dementia prevalence at baseline.

Variable	All dementia		Dementia due to AD	
	OR (95% CI)	*P* value	OR (95% CI)	*P* value
Sex (female)	1.45 (0.90–2.35)	NS	1.65 (0.93–2.92)	NS
Age	2.40 (2.02–2.85)	<0.001	1.20 (1.16–1.24)	<0.001
Education				
Illiterate	3.06 (1.21–7.77)	<0.05	2.38 (0.79–7.15)	NS
Can read and write	1.38 (0.54–3.53)	NS	1.13 (0.37–3.45)	NS
Primary school	1.63 (0.60–2.41)	NS	1.47 (0.47–4.66)	NS
≥secondary school	1 (reference)		1 (reference)	
Depression				
nD	1 (reference)		1 (reference)	
pD	1.52 (0.54–4.31)	NS	0.45 (0.06–3.40)	NS
prD	1.84 (1.01–3.35)	<0.05	1.98 (0.98–3.99)	NS
prpD	2.73 (1.08–6.87)	<0.05	3.98 (1.48–10.71)	<0.005
Hypertension	0.63 (0.41–0.96)	<0.05	0.64 (0.39–1.06)	NS
Stroke	4.11 (2.14–7.88)	<0.001	0.20 (0.02–1.60)	NS

Fully adjusted model. The variables that did not enter or did not remain in the model were diabetes, heart disease, and hypercholesterolemia.

AD: Alzheimer's disease (without vascular component); nD: never depression; pD: past depression; prD: present depression; prpD: present and past depression; NS: statistically not significant (*P* > 0.05).
